# The Physiopathological Role of IL-33: New Highlights in Bone Biology and a Proposed Role in Periodontal Disease

**DOI:** 10.1155/2014/342410

**Published:** 2014-02-18

**Authors:** Felipe Andrés Cordero da Luz, Ana Paula Lima Oliveira, Daniella Borges, Paula Cristina Brígido, Marcelo José Barbosa Silva

**Affiliations:** ^1^Institute of Biomedical Sciences, Laboratory of Immunoparasitology, Federal University of Uberlândia, Avenida Pará 1720, Building 2B, Room 2B22, CP 592, 38400-902 Uberlândia, MG, Brazil; ^2^Dentistry, Institute of Biomedical Sciences, Federal University of Uberlândia, CP 592, 38400-902 Uberlândia, MG, Brazil

## Abstract

Interleukin-33 (IL-33) is a recently described member of the IL-1 family. IL-33 acts as an alarmin, chemoattractant, and nuclear factor. ST2, a member of the Toll-like receptor/IL-1R superfamily, the receptor of IL-33, triggers a plethora of downstream effectors and leads the activation of NFK-B, leading the expression of several genes. IL-33 and ST2 are expressed in the majority of cell types, and the IL-33/ST2 axis has a role in immune response, bone homeostasis, and osteoclastogenesis. Several studies show opposite roles of IL-33 in osteoclastogenesis and the implication in bone biology. Few works studied the role of IL-33 in periodontal disease, but we hypothesize a possible role of IL-33 in periodontal disease and bone loss.

## 1. Introduction

Interleukin-33 (IL-33) is a recently described member of the IL-1 family, which includes IL-1*α*, IL-1*β*, IL-18, and IL-1Ra [1]. Endothelial and epithelial cells constitutively produce this cytokine [2]. Interleukin-33 is the ligand of the ST2 receptor, also termed T1, T1/ST2, ST2L, St2-rs1, DER4, Fit-1, Ly-84, or IL-33R, a member of the Toll-like receptor/IL-1R superfamily [3, 4].

It was initially thought that IL-33 is produced as a precursor with a molecular weight of approximately 30 kDa and is cleaved by caspase-1 to form a mature form that lacks a signal sequence, producing a protein with 20~22 kDa. However, these *in vitro* studies did not represent what in fact happens [1]. IL-33 lacks a classical caspase-1 cleavage site; a cleavage site is located in the IL-1-like domain but the cleavage products are inactive [5]. The only situation in which IL-33 is cleaved by caspases such as caspase-3 and caspase-7 is during apoptosis [5]. Also, *in vitro *assays demonstrated that 30 kDa IL-33 can be activated by neutrophil serine proteases elastase, cathepsin G, and proteinase-3 [6].Interestingly, during apoptosis, IL-33 is retained inside the nucleus, leading to its cleavage and the inactivation of its proinflammatory properties. During necrosis, IL-33 is secreted into the extracellular matrix and most likely acts as an alarmin [7]. Some studies suggest another function of IL-33 as a transcription factor. The N-terminus of this protein has a nuclear export sequence and a helix-turn-helix-like motif that can bind with heterochromatin [8]. It is thought that these properties can modulate the compaction state of chromatin, and it most likely binds to the H2A-H2B (histone 2A-histone 2B) acidic pocket of nucleosomes [9].

In earlier studies, ST2 was shown to be selectively expressed on Th2 cells but not on Th1 cells or mast cells [1]. ST2 is expressed on the surface of various cellular types, and its gene has three splicing variants, two of which are physiologically important: a soluble variant of ST2 (sST2) and a surface-attached form (ST2L) [3, 4].

Recent works have shown that IL-33 can activate murine cells by directly driving the polarization of naïve T cells to produce Th2-phenotype cells [10] and by acting as chemoattractant for human Th2 cells [11]. Additional works have demonstrated that IL-33 has a role in eosinophils [12, 13], basophils [13, 14], neutrophils [15], and macrophages [16].

IL-33 and ST2 form a complex that stimulates the recruitment of the IL-1R accessory protein (IL-1RAcp). The cytoplasmic Toll-interleukin-1 receptor domain of IL-1RAcp transduces the signal to the MyD88/IRAK1/IRAK4 (myeloid differentiation primary response gene (88)/interleukin-1 receptor associated kinase 1/4) complex and TRAF (TNF receptor associated factor), which leads to the phosphorylation of NFK-B, ERK1/ERK2 (extracellular signal-related kinase 1/2), p38, and JNK (Janus kinase) [17]. This pattern of activation leads to the production of several proinflammatory cytokines and chemokines, such as IL-5, IL-13 [1], IL-1*β*, IL-6, IL-17, INF-*γ* (interferon gamma), and TNF-*α* (tumor necrosis factor alpha) [18]. In mast cells, IL-33 activates NFK-B *via *phospholipase D and sphingosine kinase by degrading the inhibitor of kappa B (IKB) and activation of NFK-B [19], which leads to the production of several proinflammatory cytokines and chemokines [20, 21]. Importantly, IL-33 induces degranulation in IgE-primed mast cells [19] and IL-33 can induce the production of IgE in an IL4-dependent fashion in the absence of any allergen [22].  Table 1 summarizes the role of the IL-1 family members in tissue repair and osteoclastogenesis, comparing the effects of IL-33 to the other members.

## 2. The Role of Interleukin-33 in Inflammatory Bone Disease

IL-33 is a double-edged sword: while it has a protective function against parasitic infections [23–25] and sepsis [15], it also has a destructive role in several inflammatory diseases, principally allergic-type diseases [1, 26]. In the synovium of rheumatoid arthritis (RA) patients, elevated levels of IL-33 and sST2 were detected [27]. IL-33 is eminent in collagen-induced arthritis animals [28] and the inhibition of IL-33 attenuates the disease in these animals [18]. Additionally, Zaiss and colleagues performed assays in human TNF-*α*-overexpressing transgenic mice [29]. These animals develop spontaneous joint inflammation and cartilage destruction, but the administration of IL-33 inhibits the TNF-*α*-induced bone destruction *via *the IL-33/ST2 axis. Interestingly, increased levels of IL-33 in RA patients were correlated with interstitial lung diseases and bone erosion [30].

### 2.1. The Role of Interleukin-33 in Bone Homeostasis

The bone is a dynamic tissue with a complex remodeling nature. The remodeling process is orchestrated principally by three cell types: osteoblasts, osteoclasts, osteocytes, and their precursors. In physiological conditions, bone anabolism (bone formation) and bone catabolism (bone resorption) are in balance, maintaining the structure of the tissue. Bone remodeling mainly takes place adjacent to blood vessels, allowing a rapid exchange of byproducts and mediators from bone to blood and *vice versa *[31]. Additionally, bone remodeling is modulated by endocrine and paracrine mediators [32], as well as through immunological modulation by T cells [33], B cells [34], and through neural modulation [35].

Osteoblasts are derived from the embryonic mesoderm. Their differentiation is dependent on several factors. In early stages, bone morphogenic proteins (BMPs), transforming growth factor-beta (TGF-*β*), fibroblast growth factors (FGFs), and platelet-derived growth factor (PDGF) have roles in stem cell differentiation to determined osteoprogenitors. At later stages, PTH (parathyroid hormone), 1,25-(OH)_2_D_3_, insulin-like growth factor (IGF), and TGF-*β* have roles in the differentiation of determined osteoprogenitors to mature osteoblasts and eventually to osteocytes [36].

Osteoclasts are large multinucleated cells that are derived from hematopoietic monocyte/macrophage precursors. Their differentiation and activation are directly or indirectly dependent on numerous growth factors, cytokines, and hormones. These include PTH, PTHrP (parathyroid hormone related protein), IL-1, IL-6, IL-11, FGFs, IGF, RANKL (receptor activator of NFK-B ligand), M-CSF (macrophage colony stimulating factor), and TNF-*α* (tumor necrosis factor alpha). M-CSF is responsible for the differentiation of the precursors to preosteoclasts [36, 37]. RANKL, the ligand of RANK and OPG (osteoprotegerin), which is a decoy receptor, is produced by T cells and osteoblasts and is responsible for osteoclast differentiation from preosteoclasts and osteoclast activation [38]. M-CSF and RANKL are the main factors in osteoclastogenesis, as will be discussed later.

**Figure 1:**
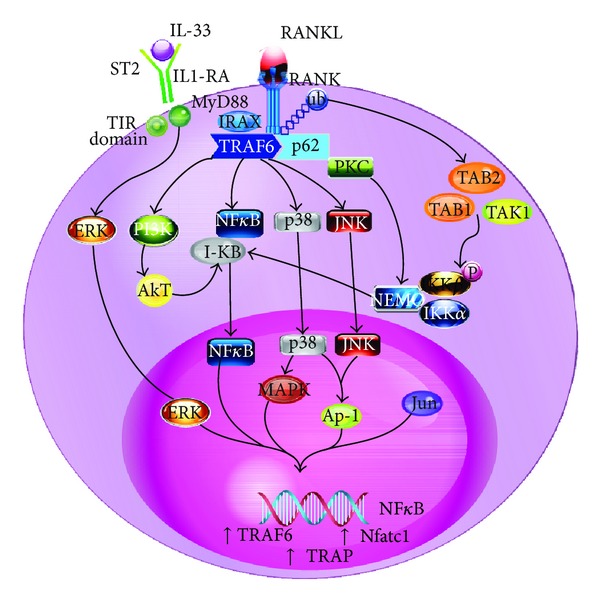
RANKL-independent signalization pathways of IL-33 and RANKL signaling in circulating monocytes. Binding of IL-33 to its receptor, ST2, leads to recruitment of MyD88 that phosphorylates ERK, activating important factors in the terminal differentiation of osteoclast as TRAF6, NFATc1, and TRAP. Activation of RANK by RANKL induces the recruitment of TRAF6, leading to the phosphorylation of PI3K, p38, and JNK. Upregulation of p62 activates PKC degrading TAB2 by ubiquitin-proteasome complex. PI3K activates AkT that phosphorylate the inhibitory IkB that is degraded by the proteasome, liberating active NFKB. The degradation of TAB2 leads to TAK1 liberation and PKC activation by p62 that, in turn, induce the phosphorylation of NEMO, liberating active NFKB. This molecule translocates to the nucleus inducing gene transcription and contributes to the activation o NFATc1. PI3K and P38 activate AP-1 that translocate to the nucleus forming a complex with NFATc1 to activate osteoclast-specific genes. The RANKL and IL-33 signalization activate intersecting downstream effectors, such as TRAF6, PI3K, JNK, and principally NFKB. Activator protein-1 (AP-1); extracellular signal-regulated kinases (ERK); inhibitor of kB (IKB); interleukin-33 (IL-33); interleukin 1 receptor antagonist (IL1-RA); interleukin-1 receptor-associated kinase 1 (IRAK); I*κ*B kinase (IKK); c-Jun N-terminal kinases (JNK); protein kinase C (PKC); mitogen-activated protein kinases (MAPK); myeloid differentiation primary response gene (MyD); nuclear factor of activated T cells (NFAT); nuclear factor kappa B cells (NFKB); receptor activator of NFKB (RANK); receptor activator of NFKB ligand (RANK-L); TAK-1-binding protein (TAB); TNF receptor associated factors (TRAF); phosphatidylinositol 3-kinases (PI3K); serine/threonine kinase (AKT); regulatory subunit NFKB-essential modulator (NEMO); tartrate-resistant acid phosphatase (TRAP); Toll-interleukin receptor (TIR); transforming growth factor-*β* activated kinase (TAK); ubiquitin (Ub).

As aforementioned, bone remodeling is also modulated by the immune system, and some researchers have studied the role of IL-33 in this context. Mun and colleagues [39] studied the differentiation of osteoclasts from human CD14^+^ monocytes, which is an osteoclast progenitor. These cells express ST2 on their surfaces, indicating a possible role of IL-33 in this process. Therefore, monocytes were cultivated in the presence of M-CSF and IL-33 or M-CSF and RANKL. Both cultures developed well-differentiated osteoclasts, as tested by TRAP (tartrate-resistant acid phosphatase, a marker of terminated differentiated osteoclasts), and the culture provided with IL-33 showed differentiation even in the presence of OPG or anti-RANKL antibody, showing RANKL-independent differentiation. Additionally, IL-33 activated all of the downstream signaling events in these cells (i.e., phosphorylation of ERK1/2, JNK, and NFK-B) and enhanced the association of NFK-B with DNA, which increased the expression of several transcription factors of osteoclast maturation, including TRAF6 (TNF receptor associated factor 6), NFATc1 (nuclear factor of activated T cells, cytoplasmic 1), c-Fos, and Syk, all of which are known to regulate osteoclast differentiation in RANKL-stimulated monocytes. Importantly, IL-33 caused the production of bone resorption factors (c-Src and cathepsin K), showing the differentiation of viable osteoclasts. It is important to note that RANKL and IL-33 increase the expression of ST2, suggesting positive feedback. Accordantly, the possible role of IL-33 in osteoclast differentiation is represented in Figure 1.

Nonetheless, other researchers have obtained different results. Saidi and colleagues [40] performed a study similar to that of Mun and colleagues. They used bone marrow stromal cells for osteoblast differentiation assays and CD14^+^ mononuclear cells from peripheral blood of healthy patients for osteoclast differentiation. First, they detected IL-33 mRNA levels in bone tissue (*ex vivo*), mainly in the bone marrow. *In vitro *assays showed expression of IL-33 in osteoblasts in these samples. Surprisingly, they found that IL-33 expression was not constitutive but was mainly inducible by TNF-*α* and IL-1*β* in these two cell samples. They incubated TNF-*α* and IL-1*β* pretreated samples of bone marrow stromal cells and CD14^+^ mononuclear cells with IL-33. They observed that IL-33 did not significantly alter the mRNA levels of ST2L when compared to the cells that had only been pretreated with TNF-*α* and IL-1*β*. These findings indicate a redundant role of IL-33 when compared to TNF-*α* and IL-1*β* in the production of ST2 *via *positive feedback. Moreover, the stimulation of CD14^+^ monocytes with IL-33 in the presence of M-CSF that was or was not associated with RANKL did not alter osteoclast differentiation, which was consistent with their finding that CD14^+^ did not constitutively express ST2L mRNA. Saidi and colleagues concluded that IL-33 is involved in the bone inflammatory status but not in normal bone homeostasis because the IL-33 and ST2L mRNA levels were increased in the presence of inflammatory cytokines. Other results suggested that IL-33 is regulated by osteogenic factors and the inhibitory effects of IL-33 may be dependent on auxiliary cells. Saleh et al. [41] demonstrated that IL-33 induced matrix mineralization deposition in primary osteoblasts. This may explain the osteogenic effects of oncostatin M and parathyroid hormone (PTH) since they induced higher expression of mRNA IL-33 in osteoblasts. Also they described that IL-33 has inhibitory effect in M-CSF/RANKL bone marrow macrophage or RAW264.7 only in the presence of osteoblast, T cells, or mature macrophages suggesting that IL-33 may act through these cells. Thus, IL-33 may not inhibit osteoclast directly in the precursor cells (preosteoclasts). Moreover, they found that the levels of IL-33 mRNA in bone marrow cells, especially osteoblast lineages, are regulated by PTH and oncostatin M.

Schulze and colleagues [42] performed assays in wild-type and ST2^−/−^ mice (ST2 null) and cell cultures. They found that osteoblasts gradually increased IL-33 mRNA expression during the course of differentiation. Osteoblasts treated with IL-33 did not alter bone formation but reduced the production of OPG and increased the production of several cytokines that influenced osteoclastogenesis (e.g., RANKL, IL-1, and IL-6). Although these data suggest an indirect role of the IL-33-osteoblast axis in osteoclast differentiation, further assays demonstrated the opposite result. By analyzing the bone mass of ST2-deficient mice, Schulze and colleagues demonstrated that the density of bone tissue decreased with higher numbers of osteoclasts, indicating an inhibitory role of IL-33/ST2 axis in osteoclastogenesis. To confirm this assertion, Schulze and colleagues performed another experiment: isolated bone marrow cells from wild-type mice did not differentiate into osteoclasts when treated with 1,25-(OH)_2_D_3_ plus IL-33. When investigating this matter, they found that IL-33 induced the expression of eosinophil and M2 macrophage gene markers and decreased osteoclastogenesis markers in these bone marrow cells. In addition, they observed that IL-33 induced its own production and enhanced the production of ST2 in these cells, suggesting a shift in cell fate during differentiation that impeded osteoclastogenesis. They also found that the inhibitory role of IL-33 in osteoclastogenesis was not due to the production of IL-4 and IL-13, cytokines that are known to inhibit osteoclastogenesis, but by IL-33-stimulated Th2 cells through the inhibition of signal transducer and activator of transcription 6 (STAT6) (a downstream mediator of the IL-4 and IL-13 signaling pathway). Assays performed in CD11b^+^ mouse leukocytes showed an inhibitory role of IL-33 in osteoclastogenesis, but not total inhibition. In summary, these findings showed a direct, antiosteoclastogenesis role of IL-33, at least in mice.

Keller and colleagues [43], who are part of the same team as Schulze, performed assays to emphasize their previous work. They induced the overexpression of IL-33 in the osteoblasts of transgenic mice and these cells were able to produce more bone matrix *in vitro*, but they did not affect bone formation *in vivo*. Importantly, no significant differences in the IL-33 serum concentrations or in the populations of eosinophils, neutrophils, basophils, or M2 macrophages of wild-type and transgenic animals were observed, indicating selective expression of IL-33 only in osteoblasts, which only impacts the bone environment. However, the most important finding was that the bone of transgenic mice showed a decreased population of osteoclasts. This was a robust finding that indicated that IL-33 inhibits osteoclastogenesis *in vivo *in mice.

Another team had similar findings. Zaiss and colleagues performed assays in human TNF-*α*-overexpressing, transgenic mice [29]. These animals developed spontaneous joint inflammation and cartilage destruction and the administration of IL-33 inhibited the TNF-*α*-induced bone destruction *via *the IL-33/ST2 axis, which was confirmed using an ST2 agonist antibody that triggered the same downstream regulators. Additionally, cartilage destruction was ameliorated by IL-33 and the osteoclast markers TRAP and NFATc1 was reduced. Importantly, the same ST2 agonist antibody was able to reduce the osteoclast population in the treated animals. They also reconstituted irradiated transgenic animals with the bone marrow of ST2^−/−^ or ST2^+/+^ animals and they observed increased bone erosion and osteoclast numbers in the animals reconstituted with bone marrow of ST2^−/−^. Treatment with IL-33 did not lead to a significant difference in the level of serum RANKL or OPG but increased the levels of IL-4, INF-*γ*, and GM-CSF. These findings show that IL-33 inhibits osteoclast formation through the production of antiosteoclastogenic cytokines, which shifts the mononuclear differentiation toward alternatively activated macrophages and dendritic cell differentiation. This group subsequently directly analyzed the role of IL-33 using osteoclast precursors (CD11b^+^) *in vitro*. Interestingly, the expression of ST2 in CD11b^+^ cells was low (10%) at the beginning of the experiment and increased (up to 70%) during osteoclast differentiation. After the addition of IL-33 at different times, in the presence or absence of RANKL or TNF-*α*, an antiosteoclastogenic role of IL-33 was revealed only at early stages by shifting the differentiation fate of the precursors. Moreover, they purified CD11b^+^ cells from human bone marrow and cultured them with RANKL and M-CSF, and the addition of IL-33 reduced the osteoclast population. In agreement with the works of Mun and Saidi, CD14^+^ peripheral blood cells are less responsive to the suppressive effect of IL-33, suggesting that in humans, as in mice, IL-33 inhibits osteoclastogenesis at early stages.

In summary, these works demonstrate the role of IL-33 in the homeostasis and the discrepancies can be explained in a simple way. An increasing number of studies suggest the existence of a highly heterogeneous subset of circulating cells in humans and mice [44]. Thus, the cells used in some works may be in a later stage of differentiation that prevents the antiosteoclastogenic role of IL-33 in the precursor cells. Notably, these works were able to detect a RANKL-independent and an indirect inhibitory role of IL-33 that stimulates the production of cytokines and shifts precursor differentiation at early stages. Importantly, the proinflammatory role of IL-33 in RA-induced animal models is mediated through mast cell degranulation [18, 28], which most likely explains the protective effect of IL-33. Finally, all of these works demonstrated constitutive expression of IL-33 in the bone, but they only studied the IL-33/ST2 axis. These studies did not demonstrate the role of IL-33 as a nuclear factor, and such strategy would most likely uncover new physiological modulation roles of IL-33 in bone homeostasis.

Additionally, these works did not consider the effects of the IL-33/ST2 axis. The inhibition of ST2 expression will affect several cells that express ST2 in their surfaces, such as mast cells, which are clearly known to trigger proinflammatory profiles. Studies of the production of IL-33-overexpressing osteoblasts have concentrated on the role of IL-33 in osteoclastogenesis, but they have disregarded the systemic effect of IL-33, thus reducing the role of IL-33 to only microenvironmental levels. It is important to state these biases, especially when studying osteoclastogenesis. As we mentioned, osteoclastogenesis is induced by proinflammatory cytokines, which are produced principally by Th1 cells and their overexpression only at microenvironmental levels conflicts with these events. We can hypothesize this because of work on TNF-*α* overexpression: TNF-*α* is a proinflammatory cytokine that induces osteoclastogenesis, and the administration of IL-33 in these animals most likely induces a Th2 profile that causes the antiosteoclastogenic effect *via *IL-4. This does not contradict the work of Shulze and colleagues, who inhibited STAT6 and observed a Th2-independent role of IL-33 in inhibiting osteoclastogenesis. A simple way to argue this is that they inhibited a downstream effect of the IL-4 and IL-13 pathways (e.g., STAT6) but not of INF-*γ*.

## 3. The Possible Role of IL-33 in Periodontal Disease 

Periodontal disease is an infectious disease that is associated with pathogenic microorganisms, which have been implicated as the primary etiologic factors in inflammatory periodontal disease. This disease affects the supporting structures of teeth, which leads to their progressive destruction and finally tooth loss. The immune system participates in the evolution of periodontitis, which indicates that bacterial antigens trigger an immunopathological reaction and that the host response to this infection is an important factor in determining the extent and severity of the disease [45].

Mast cells have an important role in this disease. Inflammation causes the destruction of tissue, including healthy tissue. After activation, mast cells express histamines, leukotrienes, prostanoids, proteases, cytokines, and chemokines, thus attracting neutrophils to the site of infection, which will ultimately eliminate the bacteria. Mast cells may participate in the pathogenesis of inflammation of periodontal disease. However, mast cells are detected in inflamed and healing gingiva. Lagdive et al. [46] stated that chronic periodontitis cases had higher mast cell counts compared to gingivitis sites or healthy tissues. Huang et al. [47] demonstrated a strong correlation between the densities of mast cells, their degranulation, and the severity of human periodontitis. Therefore, mast cell degranulation may contribute to the progression of periodontal disease.

IL-33 most likely has three roles in relation to periodontal disease: as an alarmin, a chemoattractant, and a systemic cytokine (Table 2). The release of IL-33, when acting as an alarmin, results in the destruction of several cells by necrosis, mainly fibroblasts and epithelial cells [1, 2]. In the context of inflammatory disease, IL-33 will induce other responses: mast cell degranulation and the production of proinflammatory cells (i.e., macrophages, eosinophils, and basophils). The release of inflammatory mediators and IL-33 will induce the activation of osteoblasts, which leads to the production of RANKL and diminishes the production of OPG [40] and the subsequent activation of osteoclasts. Importantly, in concordance with [39], IL-33 most likely induces osteoclast activation by increasing osteoclast transcription factors. It is well known and we have previously cited that IL-33 is involved in the anti-inflammatory response as an augmentation of the Th2 response. However, we believe that, in the context of periodontal disease, the levels of proinflammatory cytokines are extremely high due to the bacterial infection and, therefore, the osteoclastogenesis is favored. Additionally, the degranulation of mast cells in addition to the inflammation state will produce awareness of the circulating monocytes, and, in this microenvironment, they will differentiate into osteoclasts. This finding is in accordance with previously cited works that found a protective role of IL-33 inhibition in RA-induced animals [18, 28] and increased bone erosion in RA patients with IL-33 increased serum levels [30].

**Figure 2:**
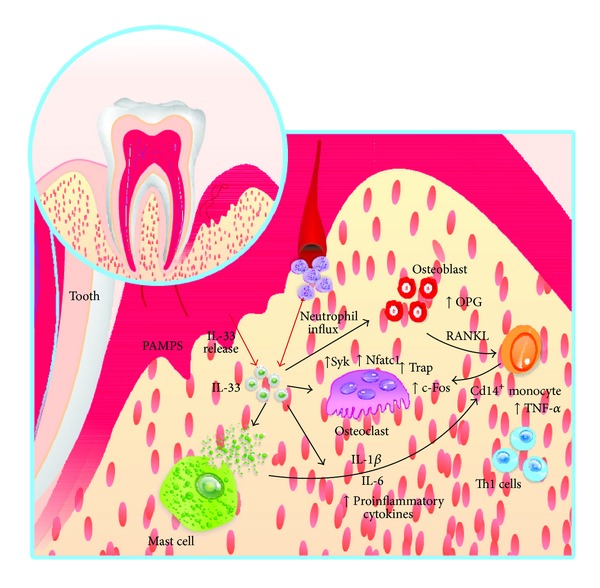
Possible role of IL-33 in periodontal disease. The destruction (necrosis) of the epithelial cells and gum releases IL-33 and acts as a chemoattractant and cytokine. The influx of mast cells, Th1 cells, and monocytes to the inflammation site, in the presence of IL-33, activates mast cell degranulation and production of proinflammatory cytokines, inducing osteoclastogenesis. Almost, IL-33 induces neutrophil influx, production of activator of nuclear factor kappa-B ligand (RANKL), and decrease of osteoprotegerin (OPG) production by osteoblast, favoring osteoclastogenesis by increasing osteoclastogenic factors as spleen tyrosine kinase (SYK), nuclear factor of activated T cells (Nfatc1), and tartrate-resistant acid phosphatase (TRAP).

Thus, we hypothesize that, in periodontal diseases, IL-33 has an osteoclastogenic role by inducing differentiation of monocytes toward proinflammatory cells that leads to the degranulation of mast cells and, therefore, the elevation of the levels of osteoclastogenic factors, thus causing osteoclast differentiation. Additionally, we believe that proinflammatory cytokines induce osteoblasts to express IL-33, which acts paracrinally (mast cells degranulation) and autocrinally (to produce RANKL and decrease OPG production). Surprisingly, according to Mun, IL-33 directly effects CD14^+^ cell differentiation in osteoclasts, and this phenomenon is independent of the RANKL-RANK axis [39] (Figure 2).

To confirm these hypotheses, it is necessary to perform at least three, large groups of experiments. One work would involve studying the osteoclastogenic potential of circulating monocytes in periodontal disease patients in the presence of TNF-*α* and RANKL with or without IL-33. We believe that monocytes from periodontal disease patients are strongly sensitized to differentiate into osteoclasts. A second work would be the study of the effects of IL-33 in circulating monocytes and bone marrow from periodontitis-induced animals, which is similar to the previously proposed work. A third work, which would be similar to the work of Schulze and colleagues, would involve analyzing the effect of the ST2 receptor, the production of proinflammatory cytokines and IL-33, osteoclast formation, and bone erosion in knockout mice with periodontitis. These works would highlight the role of IL-33 in osteoclast differentiation and/or activation in an inflamed microenvironment.

## Figures and Tables

**Table 1:** Role of IL-1 cytokine family members in tissue repair/fibrosis and osteoclastogenesis.

Common name	IL-1 family name	Cell type present	Role in tissue repair and fibrosis during inflammation	Role in osteoclastogenesis
IL-1*α* [1, 22]	IL-1F1	(i) Keratinocytes, thymic epithelium, hepatocytes, endothelial cells, fibroblasts, epithelial cells of mucus membranes (intracellular) [23] (ii) Lymphocytes or macrophages activated [23, 24]	(i) Role in the early stages of inflammation [32] (ii) Leukocyte recruitment by endothelial cells [32] (iii) Fibroblasts proliferation and collagen synthesis [32]	Activation of osteoclasts [22]

IL-1*β* [1, 22, 32]	IL-1F2	(i) Monocytes, macrophages, dendritic cells (DC), B lymphocytes, NK cells [22, 32] (ii) Gingival fibroblasts [30, 33] (iii) Macrophages, monocytes [24, 32] (iv) Th1 lymphocyte [32] (v) Osteoblasts [31]	(i) Regulates the production of metalloproteinases [30, 33] (ii) Induction and proliferation of fibroblasts and collagen [30, 33] (iii) Stimulates the release of PDGF and TGF-*β* [30, 35] (iv) Fibroblast proliferation and collagen synthesis [30, 33]	(i) Differentiation and activation of osteoclast [22, 24] (ii) Increases synthesis of collagenase [22] (iii) Increases bone resorption [22]

IL-1Ra [1, 15, 16, 27]	IL-F3	Macrophages [27]	(i) Pleiotropic actions [27] (ii) Inhibits the production of metalloproteinases (MMPs) [30, 33]	Blocks the activation of osteoclasts [31]

IL-33 [1]	IL-F11	(i) Endothelial and epithelial cells [2] (ii) Macrophages [15] (iii) Neutrophils [14] (iv) Basophiles [12, 13] (v) Eosinophils [11, 12] (vi) Th2 cells, mast cells, osteoblasts, and osteoclast [1, 19, 20]	(i) In physiological conditions are in balance, maintaining the structure of the tissue [39] (ii) Stimulates the formation of collagen and a protective function against infections [14] (iii) Produced by Th2-phenotype cells [9] (iv) Anti-inflammatory response as the augmentation of Th2 response [37]	(i) Monocyte differentiation into osteoclasts [40] (ii) Leading to the production of RANKL and diminishing the production of OPG and subsequent activation of osteoclasts [40] (iii) Inhibits the TNF-*α*-induced bone destruction via IL-33/ST2 axis [38]

**Table 2:** Role of IL-1 cytokine family members in periodontal disease.

Common name	IL-1 family name	Periodontal disease (PD) association
IL-1*α* [1, 22]	IL-1F1	(i) Increased in gingival crevicular fluid (indicative of the severity of PD) [25, 26] (ii) Associated with probing depth and attachment loss [26, 27] (iii) Influx of neutrophils and monocytes [28, 29] (iv) Stimulation of the production of PGE_2_ [30, 31]

IL-1*β* [1, 22, 32]	IL-1F2	(i) Increased in gingival crevicular fluid [26, 33] (ii) Induce the production of prostaglandin E_2_ by fibroblasts, monocytes, and macrophages [30, 33] (iii) Potentiate the effect of TNF-*α* (synergistic effect) [18, 22, 34]

IL-1Ra [1, 15, 16, 27]	IL-F3	(i) Inflammatory mediators [27] (ii) Reduction indicates severity of the PD [27]

IL-33 [1]	IL-F11	(i) IL-33 can be produced in response to bacterial presence [36] (ii) Act as “alarm,” as chemoattractant, and as a systemic cytokine [7, 25] (iii) Produce several of proinflammatory cytokines and chemokines [1, 7, 19, 20] (iv) Mast cell degranulation [37] (v) Destruction of fibroblasts and epithelial cells by necrosis [37] (vi) TNF-*α* overexpressing [38]
